# Tumour-Derived Extracellular Vesicles (EVs): A Dangerous “Message in A Bottle” for Bone

**DOI:** 10.3390/ijms20194805

**Published:** 2019-09-27

**Authors:** Alfredo Cappariello, Nadia Rucci

**Affiliations:** 1 Department of Onco-haematology IRCCS Bambino Gesù Children’s Hospital, 00152 Rome, Italy; 2 Department of Biotechnological and Applied Clinical Sciences, University of L’Aquila, 67100 L’Aquila, Italy; rucci@univaq.it

**Keywords:** Extracellular Vesicles, bone tumour, osteosarcoma, bone metastases

## Abstract

Several studies have shown the importance of Extracellular Vesicles (EVs) in the intercellular communication between tumour and resident cells. Through EVs, tumour cells can trigger cell-signalling molecules and shuttle exogenous information to target cells, thus promoting spread of the disease. In fact, many processes are fuelled by EVs, such as tumour invasion and dormancy, drug-resistance, immune-surveillance escape, extravasation, extracellular matrix remodelling and metastasis. A key element is certainly the molecular profile of the shed cargo. Understanding the biochemical basis of EVs would help to predict the ability and propensity of cancer cells to metastasize a specific tissue, with the aim to target the release of EVs and to manipulate their content as a possible therapeutic approach. Moreover, EV profiling could help monitor the progression of cancer, providing a useful tool for more effective therapy. This review will focus on all the EV-mediated mentioned mechanisms in the context of both primary bone cancers and bone metastases.

## 1. Introduction

Extracellular Vesicles (EVs) have recently become the object of intense investigation, being associated with a plethora of biological and pathological processes, although their existence has been reported for the first time in the late 40s, by Chargaff et al., who identified EVs as “small pellets sedimented by the centrifugation of the plasma at 31,000 *g*” [1,2,3,4,5,6,7,8,9,10,11,12,13,14,15,16,17,18,19,20,21]. In agreement with the International Society for Extracellular Vesicles (MISEV2018, Minimal information for studies of extracellular vesicles 2018 [22]) EVs are defined as structures surrounded by a lipidic bilayer naturally released by non-apoptotic cells [9,10]. A general classification, based on their biological characteristics, identifies two main populations: small EVs (sEVs, also known as exosomes), with less than 200 nm of diameter, and medium/large EVs (m/lEVs, also known as microvesicles) having more than 200 nm up to 1 μm of diameter. Differences in size are related to the cellular processes involved in the biogenesis of sEVs and m/lEVs. Although a consensus is still lacking about markers able to identify their origin and subtypes, different molecular mechanisms have been involved in the formation, sorting and release of EVs. Among them, the Endosomal Sorting Complex Required for Transport (ESCRT), which sorts the sEVs into an intra-cytoplasmatic bud, named MultiVesicular Body (MVB) [23]. MVBs then fuse with plasma membrane, releasing the exosomes in the extracellular environment. On the other hand, m/lEVs are generally formed by outward budding of the plasma membrane. 

EVs can be generated by different subcellular compartments, where they embed specific molecular constituents, resembling the donor cell and the compartment of origin [6,11]. The EV cargo includes nucleic acids, such as DNA, mRNAs and miRNAs, as well as cytoplasmatic and membrane-bound proteins. Once released, EVs can shuttle this multimolecular message to neighbouring cells as well as to distant targets through extracellular fluids, thus modulating their functions. 

The aim of this review is to report the most relevant evidence regarding the involvement of EVs in the onset and progression of primary bone tumours and bone metastases. Although this is still an open field which deserves to be further investigated, recent findings point out to a crucial role for EVs in the onset and development of breast and prostate cancer-induced bone metastases.

## 2. Bone and Tumours: Switching from the Virtuous to the Vicious Cycle 

Bone is a crowded but finely-regulated tissue, in which resident cells exchange with each other several biological messengers to guarantee the proper homeostasis for the skeleto [24]. The coordinated and fine-tuned orchestrated activity of osteoblasts, osteoclasts, osteocytes, endothelial cell and bone marrow resident cells could be defined as a “virtuous cycle” [25]. However, according to the theory of Paget [26], this context is also a fertile soil for the onset of both primary and secondary tumours, thus converting the virtuous cycle of the bone into a “vicious” one. Tumour cells hijack pivotal physiological bone pathways to their own advantage in order to promote their survival and proliferation, eventually leading to a suitable microenvironment for tumour growth inside the bone. As an example, osteoblast and osteocyte-released RANKL promotes tumour progression both directly, by stimulating proliferation of tumour cells through RANK, and indirectly, by promoting osteoclastogenesis, through the release of EV-bound RANKL [12,27]. 

Furthermore, both the bone and tumour need vasculature; in fact, a proper bone turnover is coupled with angiogenesis and recruitment of blood vessels [28,29], while tumour-induced neo-angiogenesis is a key event and a hallmark of tumour progression and aggressiveness. The Vascular Endothelial Growth Factor (VEGF)/VEGF receptor (VEGFR) axis is the most prominent pathway both in physiological and tumour-induced neoangiogenesis and is a therapeutic target in different types of cancers [30,31,32]. VEGF and other factors stimulating angiogenesis, such as Fibroblast Growth Factor (FGF), Platelet-Derived Growth Factor (PDGF), Interleukin (IL) 8 or regulatory miRNAs, such as miR-25-3p, are carried by tumour-released EVs [30,33,34,35,36]. 

After all, we can definitely consider the EVs as one more player through which the tumour fuels the vicious cycle in the bone microenvironment, shuttling molecules known for their effects on bone metabolism. 

## 3. Role of Extracellular Vesicles in the Progression of Primary Bone Tumours

**Figure 1:**
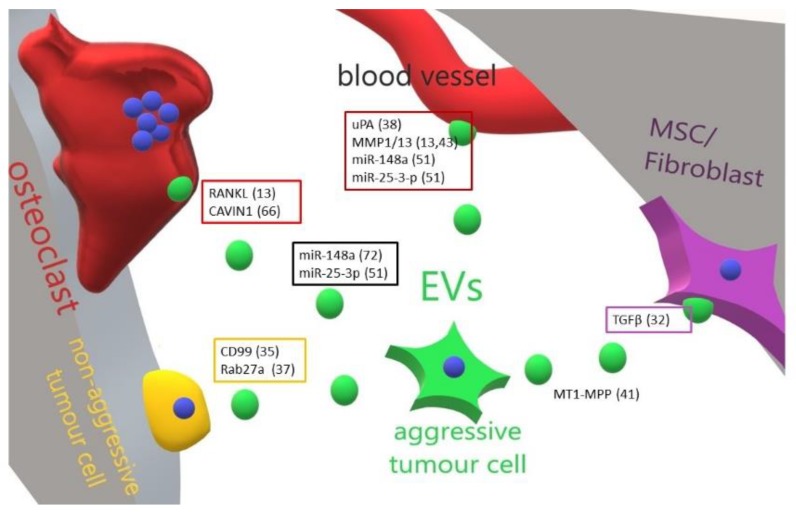
Cartoon illustrating the main effect and molecular mediators of primary bone tumour-released EVs in the bone-tumour microenvironment. An aggressive primary bone tumour cell is able to educate through EVs all the bone resident cells and to transform a cognate non-aggressive tumour cell in a metastatic one. Perturbation of bone cells homeostasis allows tumour cells to resettle in the bone and grow up. In boxes are grouped the molecules (and related references) found in EVs released by tumour cells targeting a specific cell type. ? = unknown/unspecified molecules; MSC = mesenchymal stem cells; EVs = extracellular vesicles.

Malignant bone tumours are, fortunately, rare cancers, with an incidence of around 1–2 new cases per 100,000 individuals/year [37]. Clinical features of bone tumours are often non-specific or not recognised, and as a consequence they often are not detected in the early phases [37]. Sixty percent of affected patients are younger than 45-years-old and the peak of incidence for all bone tumours occurs between 15 and 19 years. The most common primary malignant bone tumours are osteosarcoma (35%), chondrosarcoma (25%) and Ewing’s sarcoma (16%). Less than 5% of occurring tumours are fibrosarcoma of bone [37,38,39,40]. Recent papers have suggested the involvement of EVs in the pathogenesis and progression of some of these tumours [6,10,11,19,41], and many other went deeply into dissecting these effects (Figure 1, summarised in Table 1). 

Mesenchymal stem cells (MSCs) and fibroblasts often support osteosarcoma (OS) cells in bone colonisation by means of EVs exchange [42,43,44]. It is well known that the transfer of pre/mature-microRNAs between cells can be a way of intercellular communication and reprogramming of the target cells [45,46]. Furthermore, a recent paper demonstrated that EVs from OS cells can selectively shuttle a membrane form of TGFβ to MSCs, which in turn promotes the release of IL6. Accordingly, blocking the TGFβ type 1 receptor strongly decreases IL6 production in MSCs after exposition to OS-EVs [47]. Consistently, a strong association between serum levels of TGFβ and tumour progression has been found in OS patients compared to healthy individuals [47]. 

The transmembrane glycoprotein CD99, or MIC2, is an important player in the oncogenesis of Ewing sarcoma (EWS), the second most common bone tumour of children and young adults [48]. EWS is histologically characterised by lesions consisting of a core of cells showing a low-grade of neural differentiation, which is due to their neural crest origin [48,49]. Rocchi et al. found that CD99 arrests tumour cells differentiation through NFκB signalling, which in turn is regulated by a miR-34a-induced Notch pathway [50]. Silencing of CD99 in EWS cells increased miR-34a levels. Simultaneously, lower levels of Notch 1 and Notch 3 were detected in the secreted exosomes compared to exosomes released from control EWS cells [50]. Moreover, exosomes from CD99-silenced EWS cells were capable of increasing miR-34a and inhibiting NFκB signalling, with a final effect of induction of the neural differentiation of target cells [50]. Since a differentiated tumour is more favourable than an undifferentiated one, this data suggests a new therapeutic approach to EWS based on forcing the differentiation of the tumour towards a neurological phenotype.

Chemotaxis is another pivotal process promoting cancer metastasis [51]. Sung et al. showed that cancer cell chemotaxis is dependent on exosome secretion [52], since knock down of the exosome secretion regulator Rab27a inhibits the migration of fibrosarcoma cells towards a gradient of serum [52]. Moreover, the same authors found that exosome-derived fibronectin increases the speed of fibrosarcoma cells chemotaxis [52]. 

Emerging evidence suggests that cancer-derived exosomes can play a pivotal role in OS-induced metastasis. Of note, the metastatic OS cell line KHOS presents with a high expression of the urokinase Plasminogen Activator (uPA) and its plasma membrane associated receptor (uPAR), which was proved to be exclusively associated with the lung-metastatic behaviour of OS, independently from Ras status [53]. Interestingly, uPA has been detected both in exosomes and in the conditioned medium of tumours [53]. 

An increase in the production of Matrix Metalloproteinases (MMPs) is usually a general hallmark of tumour aggressiveness [54]. The extracellular enzyme Membrane Type 1 (MT1)-MMP (*alias* MMP14) plays important roles in cell migration, matrix remodelling and tumour invasion [54,55]. Extracellular vesicles isolated from human fibrosarcoma cells contain both the active full-length and inactive cleaved forms of MT1-MMP [56]. Moreover, the exosomal MT1-MMP is able to activate pro-MMP2 and to degrade type I collagen and gelatin [56]. In addition, MT1-MMP can have a synergic role in bone tumour aggressiveness due to the activation of osteoclastogenesis. In fact, one of the functions of MT1-MMP is to cleave membrane-bound RANKL, expressed on the surface of osteoblasts and osteocytes, thus inducing its release in a soluble active form, which promotes osteoclastogenesis [57]. Thus, we can conclude that exosomes are a means for MT1-MMP secretion by sarcoma cells to increase in situ aggressiveness and metastasis to lungs. Similarly, EVs were also described to contain MMP1 and MMP13 [27,58]. The involvement of MMP1 in the pathogenesis of OS has been demonstrated in the aggressive human 143B OS cell line, in which downregulation of MMP1 reduces lung metastases, while MMP1 overexpression in the less aggressive human OS HOS cells induces osteolysis and OS colonisation of lung [58]. Consistently, a direct correlation between MMP1, MMP2, MMP9 expression and poor prognosis has been reported in patients with OS [55,59,60]. In addition, tumour can take advantage directly of osteoclasts to resorb bone. In fact, OS-derived EVs were demonstrated to contain RANKL, conferring the ability to stimulate the formation and the activity of osteoclasts [27].

Tumour EVs can also be a useful tool for clinical management and screening. Proteomic analysis on EV proteins from human OS cell supernatants revealed an enrichment of proteins related to angiogenesis, cell adhesion, immune evasion and cell migration in comparison to the protein profile of exosomes from non-malignant cells [61,62]. In line with this, serum protein profile of human OS patients showed significant differences when compared to those of healthy individuals [63]. The microRNA profile also showed association with OS progression. Indeed miR-195 levels are significantly lower in sera from OS patients compared to healthy controls [64], while an increase in the miR-148a content is associated with tumour growth and metastasis [65]. miR-25-3p has also been reported to be higher in OS patients compared to controls [66]. This evidence suggests that the molecular legacy of circulating EVs could be a potential and promising clinical tool for monitoring the onset and progression of tumours, as well as an index of response to therapeutic treatments. 

## 4. Bone Metastases and Extracellular Vesicles

Solid tumours, such as breast and prostate cancers, have an undoubtable predisposition to colonise bone as secondary site, with an incidence of 70 and 90%, respectively [67]. Other primary tumours prone to colonise bone are lung, renal, colon and thyroid cancers [67].Although in breast cancer patients developing bone metastases the quality and life expectancy dramatically drop, a better prognosis is observed compared to breast cancer patients developing visceral metastases [67,68]. Moreover, among all cancer patients developing bone metastases, prostate and breast cancer patients present with the longest overall survival [69]. Although rare, bone metastases can also occur in neuroblastoma patients and, unfortunately, in this case are associated with poor prognosis [70]. 

Bone metastatic patients frequently experience a very severe symptomatology dramatically impacting the quality of life, characterised by untreatable pain, nerve compression, bone fracture, extension of the tumour into the spine and hypercalcemia, which can also cause kidney failure and cardiac arrhythmias in the final stages. All of these symptoms are called Skeletal-Related Events (SREs) and are causative of high morbidity [71]. 

Incidentally, a mention is due for haematological malignancies (HM) [72]. Indeed, although they do not cause classical bone metastases, direct and severe effects on bone is exerted from some of them, such as multiple myeloma (MM) and adult T-cell leukaemia/lymphoma (ATLL), which include osteolytic lesions leading to pathological fractures, pain and hypercalcemia. Around 80% of patients affected by MM develop SREs, with significant impact on the quality of life and the survival [73]. Many patients affected by ATLL also present with lytic bone lesions [74]. On the contrary, skeletal manifestations are quite rare in other hematological malignancies, such as Hodgkin’s and non-Hodgkin’s lymphoma [75,76]. 

**Figure 2:**
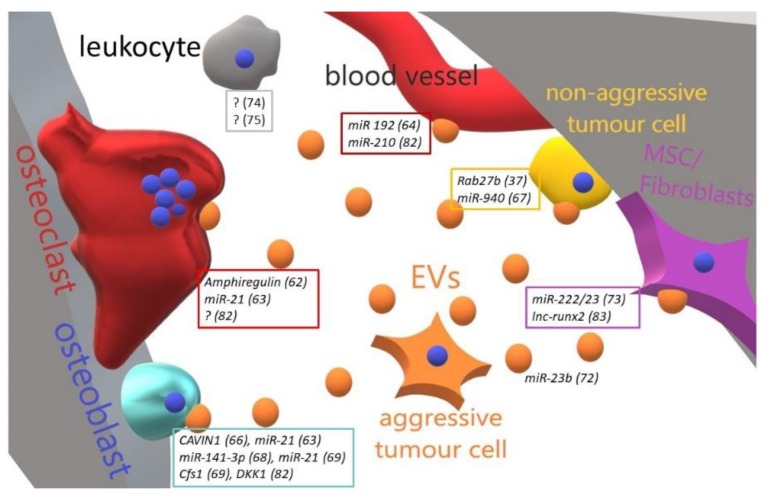
Cartoon illustrating the main effect and molecular mediators of metastatic-to-bone tumour-released EVs in the bone-tumour microenvironment. An aggressive metastatic tumour cell is able to educate through EVs all the bone resident cells and to transform a cognate non-aggressive tumour cell in a metastatic one. Perturbation of bone cells homeostasis allows tumour cells to resettle in the bone and grow up. In boxes are grouped the molecules (and related references) found in EVs released by tumour cells targeting a specific cell type. ? = unknown/unspecified molecules; MSC = mesenchymal stem cells; EVs = extracellular vesicles.

While the implication of EVs in the pathogenesis and dissemination of cancers has been well ascertained, their role in bone metastases development is still an open field, which only recently started being investigated. Results obtained so far (Figure 2, summarised in Table 2) suggest the ability of tumour-derived EVs to drive bone cells behaviour towards a microenvironment favouring tumour cells homing. Consistent with the propensity of inducing osteolytic metastases, lung cancer-derived EVs stimulate osteoclastogenesis by a mechanism requiring exosome-mediated transfer of Amphiregulin [77]. Moreover, Xu et al. found that treatment of bone marrow derived monocytes with adenocarcinoma EVs promotes osteoclast formation by shuttling miR-21, which in turn inhibits Pdcd4, an transcription factor involved in the osteoclastogenesis [78]. In contrast, Valencia et al. found that miR-192-enriched-EVs isolated from lung cancer cells act on endothelial target cells by repressing their proangiogenic programme. This result was confirmed in an in-vivo model of bone metastases, where they observed an impairment of tumour-induced angiogenesis, eventually leading to a reduction of bone metastasis growth [79].

From the side of prostate cancer-induced bone metastases, and consistent with their osteosclerotic features, Karlsson et al. showed that EVs from prostate cancer cells decrease fusion and differentiation of osteoclast precursors [80], while a previous study demonstrated that PC3 prostate derived EVs stimulate osteoblast proliferation as well as osteoclast differentiation, the latter effect being attenuated when tumour cells are transfected with a plasmid carrying cavin-1, thus pointing at this molecule as a factor that inhibits prostate cancer metastasis to bone [81]. Moreover, Hashimoto and colleagues found that hsa-miR-940 shuttled by prostate cancer EVs significantly promotes the osteogenic differentiation of human mesenchymal stem cells in vitro, by targeting ARHGAP1 and FAM134A [82]. The same authors were able to revert the osteolytic features of bone metastases induced by the osteotropic breast cancer cells MDA-MB-231 towards an osteosclerotic phenotype by overexpressing hsa-miR-940 in these cells [82]. Similar findings were observed by Ye et al., who demonstrated that metastatic prostate cancer cells- derived exosomes are incorporated by osteoblasts, where they promote their activity while reducing the osteoclast inhibitory factor OPG. This was accomplished by transferring miR-141-3p to target cells. Consistently, mice injection with exosomal miR-141-3p promotes osteoblastic bone metastases [83]. Finally, Probert et al. found that treatment with PC3-EVs increases osteoblast viability. Interestingly, this effect is blunted when EVs are isolated from Dicer-depleted PC3 cells, where miRNA biogenesis is abrogated [84].

With regards to breast cancer-induced bone metastases, recent reports also shed light on the role of EVs in the dormancy of breast cancer cells in bone marrow. The tumour dormancy is defined as a prolonged asymptomatic period during which residual cancer cells arrest proliferation and acquire drug resistance until a stimulus awake them, finally causing cancer relapse and metastasis [85,86]. It has been described that co-culturing bone metastatic MDA-MB-231 cells with bone marrow-mesenchymal stem cells (BM-MSCs) suppresses their proliferation as well as sensitivity to chemotherapy, thus suggesting the acquisition of a “dormant” phenotype by tumour cells. Interestingly, the same result was observed by culturing tumour cells with BM-MSCs-derived sEVs, due to their cargo of miR-23b [87]. Later on, Bliss et al. found that breast cancer cells can educate bone marrow mesenchymal stem cells to release sEVs, which in turn favour tumour cells quiescence and drug resistance by shuttling miR-222/223. Furthermore, systemic administration of mesenchymal stem cells loaded with antagomiR-222/223 in a mouse model of tumour dormancy sensitizes breast cancer cells to chemotherapy, eventually leading to an increase of host survival [88]. Another important aspect promoting metastasis is the ability of tumour cells to escape immune surveillance. With this regard, it has been demonstrated that breast cancer derived EVs inhibit both T-cell proliferation and NK activity in premetastatic organs including lung and liver, by promoting accumulation of myeloid-derived suppressor cells [89]. In contrast, Plebanek et al. found that sEVs isolated from the non-metastatic melanoma cells are taken up by CD11b^+^ myeloid cells, favouring the commitment towards the non-classical Ly6C^low^ monocytes, which in turn act as scavengers of tumour cells eventually leading to a reduction of metastatisation in the lung [90]. Actually, these divergences could be explained by the fact that immune cells, such as B-, T-cells and macrophages, in turn release EVs, which counteract or synergically endorse the effect of tumour EVs [14,91,92].

Hematological malignancies (HM) also take advantage of EVs to perturb bone homeostasis and induce osteolytic lesions. As an example, EVs isolated from MM stimulate osteoclastogenesis by increasing the expression of the osteoclast markers MMP9, TRAcP and Cathepsin K, as well as osteoclast migration by upregulating chemokine (C-X-C) motif receptor 4 (CXCR4) [93]. Likewise, bone marrow derived exosomes educate MM cells in a way that promotes their growth as well as drug resistance to bortezomib, a proteasome inhibitor used in MM treatment, by modulating the activation of survival pathways such as p38, p53 and Akt [94]. Other papers deeper highlight the EV involvement in the HM-related bone destruction. Kumar and colleagues showed that sEVs isolated from acute myeloid leukemia (AML) precondition the bone marrow (BM), thus accelerating AML growth [95]. AML-derived sEVs downregulate a broad range of hematopoietic stem cell-supporting factors (i.e., CXCL12, KITL and IGF1) released by BM stromal cells, thus impairing their ability to support normal haematopoiesis. On the other hands, EVs upregulate DKK1 in BM stromal cells, a suppressor of normal hematopoiesis and osteogenesis in bone marrow [95]. In fact, inhibiting the sEVs secretion in AML cells by targeting Rab27a, an important regulator of sEVs release, significantly delays leukaemia development. Moreover, DKK1 is shuttled by MM derived EVs and transferred to osteoblasts, where it impairs Runx2, Osterix, and Collagen 1A1 expression [96]. Consistently, a synergic inhibitory effect of bortezomib plus the sEV inhibitor GW4869 on MM in vivo growth has been observed [96]. A further dissection of these pathways has been performed by Li et al., demonstrating that cells from MM patients are enriched in long non coding (lnc) RNA against Runx2 pre-mRNA (lncRUNX2-AS1) which is shuttled through MM derived sEVs and transferred to MSCs, eventually decreasing osteogenesis [97]. Finally, Raimondo et al. demonstrated that exosomes isolated from MM cells collected from BM aspirates shuttle the EGFR ligand Amphiregulin (AREG), which is responsible for the sEV-induced osteoclastogenesis [98].

## 5. Therapeutic Perspectives of Extracellular Vesicles

Due to their involvement in cancer propagation and metastasis, EVs have been investigated as promising therapeutic tools to counteract tumour progression. In this context, two potential strategies can be envisaged: (i) targeting the release of cancer EVs, avoiding the spread and progression of the tumours or (ii) using EVs as natural carriers for drug/gene delivery.

### 5.1. Targeting the Release of Cancer-Derived EVs

Due to their ability to “educate” target cells once released, the first suitable strategy to counteract the pro-tumoural effect of EVs could be blocking those cellular pathways involved in EVs biogenesis, thus impairing their release. In line with this strategy, a potential target is ceramide, one of the lipids identified in the ESCRT-independent biogenesis pathways and synthesized by neutral sphingomyelinase 2 (nSMase2) [7,99]. Perturbation in ceramide production has been pursued either by knocking down nSMase2 or treating with its inhibitor GW4869 [99,100,101]. In line with this, Kosaka and colleagues demonstrated that MDA-MB-231-derived sEVs shuttle miRNA-210, which in turn affect endothelial cells and increase lung metastasis [102]; however, this effect was abrogated by knocking down sSMase2 in breast cancer cells [102]. Another study by Singh et al. showed that the highly invasive MDA-MB-231 cells express higher levels of miR-10b compared to the less aggressive MCF7 cells and to the human mammary epithelial cell line HMLE [103]. miR-10b released by MDA-MB-23-EVs reduces Homeobox D10 (HOXD10) expression, thus promoting invasion ability and proliferation in target HMLE cells, however this effect is blunted when treating MDA-MB-231 with GW4869 [103].

Another approach to interfere with the secretion of EVs is targeting the Rab proteins, which are crucial for sEVs biogenesis [104,105,106]. They are small GTPase belonging to Ras GTPases family, known to be key regulators of the intracellular trafficking [104,105,106]. In particular, Rab 27a and 27b are involved in MVB docking and exocytosis and their knocking down in HeLa cells by RNA interference reduces EVs release [104]. Similar results were obtained silencing two Rab27 effectors, Slp4, *alias* synaptotagmin-like 4 (SYTL4) and Slac2b (i.e., exophilin 5, EXPH5) [104]. Furthermore, short hairpin-mediated (sh) Rab 27a inhibition in the 4T1 breast carcinoma cell line results in a reduction of tumour growth and metastasis [107]. Another important member involved in EVs biogenesis is Rab 11, playing a role in docking and fusion of MVBs, as well as in calcium-dependent sEVs release [106]. A mutant form of Rab11, lacking GTPase activity, leads to a reduction of calcium-mediated sEV release in the human erythroleukemia cell line K562 [106].

On the other side, blocking EVs uptake and internalization has been exploited to stop tumour progression [108,109,110]. EVs internalization is achieved by different mechanisms, such as clathrin-mediated endocytosis, phagocytosis, micropinocytosis, and plasma or endosomal membrane fusion, [110]. Moreover, adhesion and internalization of sEVs can require the interaction with Heparan Sulfate (HS) proteoglycans (HSPGs) proteins [109]. Christianson et al. showed that treatment with the HS mimetic heparin inhibits sEV uptake in glioblastoma cells with a consequent reduction of their migration [109]. Another suitable approach is based on the inhibition of the clathrin-mediated endocytosis pathway. Macia et al. identified an inhibitor of dinamin1/2 activity, called dynasore, able to block clathrin coats formation on the plasma membrane [108]. Some years later, Kawamoto demonstrated that dynosore blocks the uptake of human melanoma cells-EVs by endothelial cells, thus preventing their transformation in tumour-associated endothelial cells (TECs) [20]. Similar results were found with Mantle Cell Lymphoma (MCL), an aggressive mature B cells neoplasm [111]. The authors found that sEVs from MCL patients were uptaken by healthy B-cell derived patients through a cholesterol-dependent mechanism [111]. The use of dynasore inhibits the uptake of MCL-released EVs in both healthy and MCL B-lymphocytes, confirming that EVs internalization occurs through the micropinocytosis, being this process dynamin independent [111].

### 5.2. EVs as Biological Vehicles for Active Molecules

Due to their nature, EVs could be optimal vehicle to deliver drugs or nucleic acids agents, providing several advantages compared to other methods of shuttling. In fact, EVs are stable under both physiological and pathological conditions, since they are naturally present in body fluids [112,113,114]. Additionally, EVs are selective carriers to specific recipient cells, due to the unique membrane proteins and lipids that can bind to specific receptors expressed by target cells, enhancing the delivery efficiency [115]. For these reasons, EVs are nowadays tested for both gene therapy and drug vehicle in different fields. Gene therapy as anti-cancer strategy is generally hard to apply, due to genome heterogeneity and instability of the tumour, however one interesting application has been proposed by Mizrak et al. [116]. The authors introduced into HEK293T cells a “suicide gene” encoding for a chimeric protein formed after in frame-fusion of cytosine deaminase (CD) with uracil phosphoribosyl transferase (UPRT). This suicide gene promotes the conversion of 5-fluorocytosine (5-FC) to 5-fluorouracil (5-FU), which in turn is converted in 5-fluoro-deoxyuridine monophosphate (5-FdUMP), an irreversible inhibitor of thymidine synthetase that is more toxic for cancer cells than 5-FU. EVs isolated from engineered HEK293T cells are able to deliver the suicide gene/protein into the mouse sciatic nerve schwannomas model, resulting in a significant increase of the antitumoral effect of this chemotherapeutic [116].

The small size of EVs, especially sEVs, and the long half-life in the circulation make them an ideal drug vehicle [117]. To this purpose, Tian and colleagues loaded by electroporation the sEVs isolated from mouse immature dendritic cells (imDCs) with the chemotherapeutic doxorubicin (DXR) [118]. The imDCs were also engineered to express the exosomal membrane protein Lamp2b fused to αv integrin-specific iRGD peptide (CRGDKGPDC) in order to increase the interaction with αv expressing target cells, such as MDA-MB-231. The iRGD DXR-sEVs proved to be efficacious in reducing MDA-MB-231 cells in an orthotopic mouse model of breast cancer, showing higher efficiency and less toxicity compared to free DXR [118].

## 6. Conclusions

The identification of extracellular vesicles as crucial mediators of the cell-cell exchange of biological information has complicated the scenario of tumour-induced vicious cycle in bone. Tumour-derived EVs educate bone resident cells to promote their survival and growth. Therefore, a better understanding of the biology of EVs could help opening alternative strategies to fight bone colonisation by tumour cells. However, there are several challenges that still need to be addressed. As a matter of fact, a major limitation in this field is the lack of well standardized methods for EVs isolation and purification. So far, the most employed method is ultracentrifugation, which, however, is time-consuming, and the obtained EVs are often a mix of different subtypes of EVs, being a limitation for those researchers interested to a specific subset of EVs. Indeed, most of the studies focused on drug/gene delivery employ sEVs due to a small size and a lower complexity of the molecular cargo. Developing a faster and more selective method of exosomes isolation is therefore one of the most important tasks in the current field of research.

Current cell culture and EVs purification technologies restrict the implementation of standardized and mass production of EVs [119]. Therefore, in order to speed up the research toward the pathological mechanisms regulated by EV and their employ in the therapeutic platform, scalable manufacturing processes should be identified to isolate EVs in a fast, reproducible and cost-effective way.

Extracellular cell source should be carefully identified according to the specific applications. For example, in the perspective of utilizing sEVs for anti-cancer therapeutics, cancer cell- sEVs should be obviously avoided, as they could contain oncogenic drivers contributing to cancer progression. However, some authors proposed to use cancer EVs as theranostic tool. As an example, Hazan-Halevy et al. proposed that their results on selective uptake of MCL EVs (discussed above in Section 5.1) could be transposed in potential clinical strategies. In particular, the authors speculate that MCL-EVs isolated from patient’s serum could be manipulated by labelling with imaging probes and administered back to the same patient in order to selectively trace the tumour [111]. On the other hands, the same EVs could be loaded with a therapeutic cargo and injected back to specifically hit the cancer cells [111]. Similarly, other authors set the basis for the use of tumour-EVs as “antigens” for the production of a cancer “vaccine”, educating the immune cells to fight cancer cells [120,121].

In conclusion, thorough and precise characterisation studies of EVs are still needed to standardize the methods and clarify the biological effects of EVs, both in physiological and pathological conditions. Therefore, a rigorous methodology and characterisation will open the avenue for a better employ of EVs as therapeutic carriers and diagnostics tools.

## Figures and Tables

**Table 1:** List of molecular educators, cell targets and effects of EVs isolated from primary bone tumours.

Tumour	Educator	Target Cell	Effect	Ref.
Osteosarcoma	TGF beta	MSCs	Increase of IL-6 production	[47]
Ewing Sarcoma	CD99	Autologous	Increase of miR-34a Inhibition of NFκB	[50]
Fibrosarcoma	Rab27a	Autologous	Induction of chemotaxis	[52]
Osteosarcoma	uPA	Lung	Pro-metastatic	[53]
Fibrosarcoma	MT1-MMP	Extracellular matrix	Activation of MMP2 Degradation of type 1 collagen and gelatine	[56]
Osteosarcoma	MMP1/MMP13	Lung	Increase of aggressiveness	[27] [58]
Osteosarcoma	RANKL	Osteoclasts	Increase of osteoclastogenesis	[27]
Osteosarcoma	miR-148a	Unspecified	Increase of aggressiveness	[66]
Osteosarcoma	miR-25-3-p	Unspecified	Increase of aggressiveness	[66]

**Table 2:** List of molecular educators, cell targets and effects of EVs isolated from metastatic bone tumours.

Tumour	Educator	Target Cell	Effect	Ref.
Lung cancer	Amphiregulin	Osteoclasts	Increase of osteoclastogenesis	[77]
Lung cancer	miR-21	Osteoclasts	Increase of osteoclastogenesis	[78]
Lung cancer	miR-192	Endothelial cells	Increase of osteoclastogenesis	[79]
Prostate cancer	Unspecified	Osteoclasts	Inhibition of osteoclast formation, activity and survival	[80]
Prostate cancer	Cavin1	Osteoblasts	Inhibition of osteoblast proliferation	[81]
Prostate cancer	Cavin1	Osteoclasts	Inhibition of osteoclast differentiation	[81]
Prostate/Breast cancer	hsa-miR-940	Autologous	Inhibition of metastasis	[82]
Prostate cancer	miR-141-3p	Osteoblasts	Increase of osteoblast activity, decrease of OPG expression	[83]
Prostate cancer	miRNA (i.e., miR-21) and mRNA (i.e., CSF-1) pool	Osteoblasts	Increase of osteoblast viability	[84]
Breast cancer	miR-23b	HSC	Dormancy induction	[87]
Breast cancer	miR-222/23	MSC	Dormancy induction	[88]
Breast cancer	Unspecified	Myeloid, T- and NK cells	Inhibition of immune response	[89]
Multiple Myeloma	Dkk1 Unspecified(?)	Osteoblasts, Osteoclasts	Decrease of osteoblast differentiation, increase of osteoclast activity	[96]
Multiple Myeloma	lncRNA vs RUNX2	MCS	Silencing of RUNX2 mRNA and decrease of osteogenesis	[97]
